# Functional Therapeutic Target Validation Using Pediatric Zebrafish Xenograft Models

**DOI:** 10.3390/cancers14030849

**Published:** 2022-02-08

**Authors:** Charlotte Gatzweiler, Johannes Ridinger, Sonja Herter, Xenia F. Gerloff, Dina ElHarouni, Yannick Berker, Roland Imle, Lukas Schmitt, Sina Kreth, Sabine Stainczyk, Simay Ayhan, Sara Najafi, Damir Krunic, Karen Frese, Benjamin Meder, David Reuss, Petra Fiesel, Kathrin Schramm, Mirjam Blattner-Johnson, David T. W. Jones, Ana Banito, Frank Westermann, Sina Oppermann, Till Milde, Heike Peterziel, Olaf Witt, Ina Oehme

**Affiliations:** 1 Hopp Children’s Cancer Center Heidelberg (KiTZ), 69120 Heidelberg, Germany; charlotte.gatzweiler@dkfz-heidelberg.de (C.G.); j.ridinger@kitz-heidelberg.de (J.R.); sonja.herter@kitz-heidelberg.de (S.H.); xenia.gerloff@dkfz-heidelberg.de (X.F.G.); d.elharouni@kitz-heidelberg.de (D.E.); yannick.berker@kitz-heidelberg.de (Y.B.); r.imle@kitz-heidelberg.de (R.I.); lukas.schmitt@dkfz-heidelberg.de (L.S.); s.kreth@kitz-heidelberg.de (S.K.); s.stainczyk@kitz-heidelberg.de (S.S.); s.ayhan@kitz-heidelberg.de (S.A.); s.najafi@kitz-heidelberg.de (S.N.); p.fiesel@kitz-heidelberg.de (P.F.); kathrin.schramm@kitz-heidelberg.de (K.S.); m.blattner-johnson@kitz-heidelberg.de (M.B.-J.); david.jones@kitz-heidelberg.de (D.T.W.J.); a.banito@kitz-heidelberg.de (A.B.); f.westermann@kitz-heidelberg.de (F.W.); sina.oppermann@kitz-heidelberg.de (S.O.); t.milde@kitz-heidelberg.de (T.M.); h.peterziel@kitz-heidelberg.de (H.P.); o.witt@kitz-heidelberg.de (O.W.); 2 Clinical Cooperation Unit Pediatric Oncology, German Cancer Research Center (DKFZ) and German Cancer Consortium (DKTK), 69120 Heidelberg, Germany; 3 Faculty of Medicine, Heidelberg University, 69120 Heidelberg, Germany; 4 Faculty of Biosciences, Heidelberg University, 69120 Heidelberg, Germany; 5 Faculty of Mathematics and Computer Science, Heidelberg University, 69120 Heidelberg, Germany; 6 Bioinformatics and Omics Data Analytics, German Cancer Research Center (DKFZ), 69120 Heidelberg, Germany; 7 Division of Pediatric Neurooncology, German Cancer Research Center (DKFZ) and German Cancer Consortium (DKTK), 69120 Heidelberg, Germany; 8 Pediatric Soft Tissue Sarcoma Research Group, German Cancer Research Center (DKFZ), 69120 Heidelberg, Germany; 9 Department of General, Visceral and Transplantation Surgery, Division of Pediatric Surgery, University Hospital Heidelberg, 69120 Heidelberg, Germany; 10 Division of Neuroblastoma Genomics, German Cancer Research Center (DKFZ), 69120 Heidelberg, Germany; 11 Department of Pediatric Oncology, Hematology and Immunology, Heidelberg University Hospital, 69120 Heidelberg, Germany; 12 Light Microscopy Facility, German Cancer Research Center (DKFZ), 69120 Heidelberg, Germany; d.krunic@dkfz-heidelberg.de; 13 Institute for Cardiomyopathies Heidelberg, Heidelberg University, 69120 Heidelberg, Germany; karen.frese@med.uni-heidelberg.de (K.F.); benjamin.meder@med.uni-heidelberg.de (B.M.); 14 Genome Technology Center, Stanford University, Stanford, CA 94304, USA; 15 Department Neuropathology, Institute of Pathology, Heidelberg University Hospital, 69120 Heidelberg, Germany; david.reuss@med.uni-heidelberg.de; 16 Clinical Cooperation Unit Neuropathology, German Cancer Research Center (DKFZ), 69120 Heidelberg, Germany; 17 Division of Pediatric Glioma Research, German Cancer Research Center (DKFZ) and German Cancer Consortium (DKTK), 69120 Heidelberg, Germany

**Keywords:** zPDX, mPDX, functional precision oncology, drug screen, patient-derived spheroid culture, small molecule inhibitors, targeted therapy

## Abstract

**Simple Summary:**

Despite the major progress of precision and personalized oncology, a significant therapeutic benefit is only achieved in cases with directly druggable genetic alterations. This highlights the need for additional methods that reliably predict each individual patient’s response in a clinically meaningful time, e.g., through ex vivo functional drug screen profiling. Moreover, patient-derived xenograft (PDX) models are more predictive than cell culture studies, as they reconstruct cell–cell and cell–extracellular matrix (ECM) interactions and consider the tumor microenvironment, drug metabolism and toxicities. Zebrafish PDXs (zPDX) are nowadays emerging as a fast model allowing for multiple drugs to be tested at the same time in a clinically relevant time window. Here, we show that functional drug response profiling of zPDX from primary material obtained through the INdividualized Therapy FOr Relapsed Malignancies in Childhood (INFORM) pediatric precision oncology pipeline provides additional key information for personalized precision oncology.

**Abstract:**

The survival rate among children with relapsed tumors remains poor, due to tumor heterogeneity, lack of directly actionable tumor drivers and multidrug resistance. Novel personalized medicine approaches tailored to each tumor are urgently needed to improve cancer treatment. Current pediatric precision oncology platforms, such as the INFORM (INdividualized Therapy FOr Relapsed Malignancies in Childhood) study, reveal that molecular profiling of tumor tissue identifies targets associated with clinical benefit in a subgroup of patients only and should be complemented with functional drug testing. In such an approach, patient-derived tumor cells are exposed to a library of approved oncological drugs in a physiological setting, e.g., in the form of animal avatars injected with patient tumor cells. We used molecularly fully characterized tumor samples from the INFORM study to compare drug screen results of individual patient-derived cell models in functional assays: (i) patient-derived spheroid cultures within a few days after tumor dissociation; (ii) tumor cells reisolated from the corresponding mouse PDX; (iii) corresponding long-term organoid-like cultures and (iv) drug evaluation with the corresponding zebrafish PDX (zPDX) model. Each model had its advantage and complemented the others for drug hit and drug combination selection. Our results provide evidence that in vivo zPDX drug screening is a promising add-on to current functional drug screening in precision medicine platforms.

## 1. Introduction

The prognosis of children with high-risk and relapsed tumors remains poor with an overall survival rate of less than 20% after two years, despite multi-modal treatment regimens [1,2]. The INFORM pediatric precision oncology program (INdividualized Therapy FOr Relapsed Malignancies in Childhood) tackles this unmet medical need by implementing a molecular diagnostic next-generation sequencing (NGS) pipeline and a clinical follow-up of pediatric patients. INFORM is an international registry study, with more than 2000 enrolled cases from over 100 pediatric oncology centers in 13 countries [3]. Results from this study demonstrated that progression-free survival could be doubled in a subgroup of patients with very high evidence targets receiving a molecularly matched targeted treatment [4]. As patients with low evidence molecular targets failed to benefit from the molecularly matched targeted treatment approach alone, the INFORM study was amended with drug sensitivity profiling and the establishment of three-dimensional cultures from viable tumor samples.

Functional precision oncology aims at meeting the patient’s needs by identifying promising treatment options for every cancer and patient individually with ex vivo functional assays. As an add-on to genomic data, functional studies such as ex vivo 3D cell cultures aim to further improve and tailor drug treatment for each individual patient [5]. Three-dimensional cell growth is known to show differences in gene transcription, cell cycle and population doubling time, metabolism as well as cell death and differentiation compared to two-dimensional monolayer cultures [6]. Moreover, 3D-grown tumor cells display enhanced resistance to drug treatment, thereby more closely reflecting the in vivo situation [7]. Ideally, however, to reliably predict the individual patient response in a clinically meaningful manner, patient-derived tumor cells should be exposed to suitable approved oncological drugs in a more physiological setting (compared to cell culture experiments), which also reflects the interaction with the surrounding tumor microenvironment (TME). This can be achieved by testing a panel of drugs on animal avatars of patient tumor cells to identify the most promising treatment for this particular patient.

Patient-derived xenograft (PDX) models are considered to be more predictive than cell culture studies, as they reconstruct cell-–ell and cell–extracellular matrix (ECM) interactions, which enable cell polarization, differentiation, and migration [8]. Although non-humanized PDX models lack a functional immune system, they can recapitulate cellular and acellular components of the tumor microenvironment, which influence tumor cell behavior and therapy sensitivity. While mouse PDXs are the most widely used model, zebrafish (Danio rerio) PDXs are also emerging as a cheaper and much faster model allowing many drugs and combinations to be tested at the same time [9].

In recent years, the engraftment of human cancer cells into zebrafish has led to the identification of promising novel treatment options [10,11,12,13,14,15,16]. Zebrafish early larvae are optically transparent, and their adaptive immune system is immature during the first weeks, which makes them a very suitable model for cell engraftments and live imaging [17,18,19]. The yolk sac, one of the most widely used injection sites in zebrafish early larvae, offers a nutrient-rich environment for transplanted cells. The model needs only a few hundred implanted cells per embryo, which makes it an attractive model when working with primary patient-derived tumor material. 

Here, we aimed to further optimize precision medicine approaches by adding zebrafish PDX (zPDX) models to the molecular and functional drug screening pipeline. By using tumor samples from the INFORM study, we first compared the molecular tumor profile and the drug screening results of patient-derived spheroid cultures shortly after tumor dissociation, reisolated cells from corresponding mouse PDX tumors and corresponding long-term organoid-like cultures, and then used one culture for the zPDX model for further drug hit and drug combination evaluation in vivo. We here show that ex vivo drug screens in combination with in vivo zPDX validation models are a promising add-on in precision oncology to improve cancer treatment.

## 2. Materials and Methods

### 2.1. Primary Cell Isolation from Fresh Tumor Tissue

Fresh surgical specimens of patients obtained through the INFORM study (INdividualized Therapy FOr Relapsed Malignancies in Childhood [3]) were mechanically and enzymatically dissociated according to a protocol adapted from Stewart et al. [20]. Briefly, the tumor tissue was chopped into small pieces with a sterile scalpel, and the soft tissue sarcoma and the rhabdoid tumor samples (INF_R_1288_relapse1 and INF_R_1467_relapse1) were subsequently digested with 120 µg/mL trypsin (T9935, Sigma-Aldrich, Munich, Germany) and 1mg/mL collagenase II (17101015; Thermo Fisher Scientific, Waltham, MA, USA) in DMEM (without supplements) for 60 min at 37 °C with repeated mixing every 10 min. The neuroblastoma sample (INF_R_359_relapse3) was digested with 120 µg/mL trypsin in Neurobasal A (10888022; Life Technologies) for 10 min at 37 °C. The enzymatic reactions were stopped by adding 120 µg/mL of trypsin inhibitor (T6522, Sigma Aldrich). Free DNA released from dead cells was removed by repeated addition of 1 mg/mL DNAse in 0.5 M MgCl_2_. The cell suspensions were filtered through 70 µm Falcon^®^ cell strainers (352350; Corning, Corning, NY, USA), followed by red blood cell lysis with ACK Lysing Buffer (A1049201; Thermo Fisher Scientific) according to the manufacturer’s protocol. Cells were washed two to three times with TSM base and resuspended in TSM complete at a density of 2–3 × 10^6^ cells per ml, resulting in the formation of free-floating three-dimensional spheroids and semi-adherent spheroids within 24 h after tumor dissociation (fresh tissue culture, FTC).

Long-term culture establishment was performed as described previously [21]. Briefly, when reaching a spheroid diameter of approx. 700–1000 nm, cells were sub-cultured by dissociation with TrypLE express (12604013; Life Technologies, Carlsbad, CA, USA) and seeding at a ratio of 1:2 to 1:5 in fresh TSM complete. The cells were considered as established long-term cell cultures (LTC) upon reaching ex vivo passage 6. The term ‘long-term’ was chosen to distinguish the at least six times passaged cultures from non-passaged fresh tissue cultures (FTCs), which were used within the first week of culture. The absence of mycoplasma, SMRV or interspecies contamination was confirmed by Multiplex cell Contamination Test (McCT) by Multiplexion (Heidelberg, Germany) as described previously [22]. The cells were cultured serum-free as spheroid cultures in TSM complete medium, containing Neurobasal A Medium, DMEM F-12, Hepes Buffer, Sodium Pyruvate, MEM NEAA, L-Glutamine, Penicillin/Streptomycin (Sigma-Aldrich), B-27 supplement (Gibco/ThermoFisher, Braunschweig, Germany), EGF, FGF, PDGF and Heparin (0.2%).

### 2.2. Tumor Cell Isolation from Mouse PDX

In addition to the establishment of LTC, approximately 1–6 x 10^6^ cells from each tumor sample were subcutaneously injected into immunocompromised NOD/scid/gamma (NSG) mice immediately after tumor cell isolation to generate murine patient-derived xenograft (mPDX) models. Mouse tumors were dissociated and re-transplanted into further recipient mice over a total of four passages to regard the model as stably established. Cells were viably frozen for re-injection. Tumor-bearing mice were sacrificed when the volume of the subcutaneous tumor reached approximately 400–1400 mm^3^. Tumor cell isolation from the PDX tumors was performed according to the same protocol as described above for the respective fresh tumor samples.

### 2.3. Molecular Diagnosis

For both the LTC and the cultures established from PDX tumors (PDX-C), the molecular diagnosis and maintenance of methylation profile and copy number aberrations were assessed with the Infinium MethylationEPIC Bead Chip (Illumina, San Diego, CA, USA) according to the manufacturer’s instructions. The data were used for molecular classification by comparison with an in-house reference set for sarcomas [23] and an in-house reference set for high-risk (HR) MYCN amplified neuroblastomas [24] and compared to the respective data of the original tumor.

### 2.4. Drug Screening and Metabolic Activity Assays

Cells were seeded to form 3D spheroids in ultra-low attachment 384-well plates (3830, Corning, Corning, NY, USA, U-bottom) in 20 µL TSM medium per well (Table 1). The drugs were administered to the plates before seeding and consist of at least 76 clinically relevant anti-cancer drugs (targeted as well as chemotherapy) by the Institute for Molecular Medicine Finland (FIMM) High Throughput Biomedicine unit (FIMM, Helsinki Institute of Life Science, University of Helsinki, Finland) with the Echo 550 OMICS2 (Labcyte, San José, CA, USA) or at KiTZ by using the TECAN D300e and the Mosquito (SPT Labtech, Melbourn, UK). Cell viability was quantified after 72 h in culture using CellTiter-Glo^®^ 2.0 kit (Promega, Madison, USA). Specifically, 15 µL CellTiter-Glo^®^ (384-well) was added to every well, plates were shaken at 400 rpm for 5 min and afterwards incubated for another 10 min. Bioluminescence was read on a TECAN Spark (TECAN, Männedorf, Switzerland) or PheraStar (BMG Labtech, Ortenberg, Germany) plate reader. Results were analyzed for dose–response curves and drug sensitivity with the iTReX Shiny app [25] (LTCs and PDX-Cs; DSS_asym_ computed with A_min_ set to 10% from I_max_) or an in-house developed automated drug screen analysis pipeline (FTCs).

### 2.5. Zebrafish Lines

The care for and breeding of the zebrafish were performed under standardized conditions, as described previously [26]. Zebrafish wild-type AB line was raised at 28 °C. Embryos used for tumor injections were maintained in an E3 buffer (292.2 mg/L NaCl; 12.6 mg/L KCl; 36.3 mg/L CaCl_2_; 39.8 mg/L MgSO_4_) supplemented with 0.2 mM 1-phenyl-2- thiourea (PTU, Sigma).

### 2.6. Zebrafish Embryo Toxicity Assays

The toxicity of the drugs of interest (targeted therapy and chemotherapy) was assessed in vivo before performing in vivo tumor growth experiments. The maximum tolerated dose (MTD) was determined as the concentration where embryos displayed no signs of toxicity, such as death or morphological changes (curvature of body, edema). Specifically, 48 hpf embryos were transferred to uncoated 48-well plates (Corning) containing assay buffer, supplemented with increasing concentrations of the drug of interest and the corresponding solvent control. To reach the final concentration in the assay buffer, the stock solution of the compounds was diluted in E3 buffer supplemented with PTU. Three embryos were tested per concentration or solvent control. Embryos were held at 34 °C during the experiment and imaged at 72 hpf and at 120 hpf using a stereo microscope (Leica, Biosystems, Wetzlar, Germany).

### 2.7. Cell Preparation for Zebrafish Embryo Xenotransplantation

Spheroids were dissociated into single cells with TrypLE express (Life Technologies, Thermo Fisher Scientific Inc., Waltham, MA, USA; 5 min, 37 °C) as described previously [13]. To determine the number of viable cells/mL, 1 mL of cell suspension was used with the automated ViCell cell counter, which uses trypan blue to discriminate viable from dead cells.

Next, 1 × 10^6^ viable cells/mL were stained with 5 µL of CM-DiI (CellTracker CM-DiI; Thermo Fisher Scientific, Waltham, MA, USA) solution, according to the manufacturer’s instructions. Briefly, cells were incubated at 37 °C for 10 min, then centrifuged and washed three times with FCS-free RPMI medium without phenol red. Cells were resuspended in 1 mL of the same medium and incubated with 1 µL of Benzonase for 10 min at room temperature. Cells were centrifuged once more and resuspended to 1.5 × 10^6^ cells/10 µL. For INF_R_359 cells, a 40 µM cell strainer was used before labeling the cells, in addition to the steps described before.

For the duration of injections, cells were kept on ice and embryos were anesthetized with tricaine (MS-222, tricaine methanesulfonate, 0.02% (*w/v*); Sigma-Aldrich, Munich, Germany; 1 × tricaine) prior to the injection. Microinjection needles (Science Products, Hofheim, Germany) were pulled using a Capillary puller (Sutter instruments, Novato, CA, USA). The tip of the pipette was cut with a scalpel under the microscope to obtain a capillary opening that fit to the size of the cells. Approximately, 8–10 µL of the cell suspension were filled into one capillary and injected into the yolk sac of the embryos using the Eppendorf FemtoJet express microinjector (Eppendorf, Hamburg, Germany) and Eppendorf Micromanipulator. Tumor cell injected embryos were kept at 34 °C and tumor cell fluorescence in the yolk sac was checked via fluorescence imaging after 1–2 hpi (hours post-injection). On average approximately 70–80% of embryos were successfully injected with fluorescently labeled LTC cells. The zPDX were sorted into groups with the same mean tumor size, each group containing some bigger, some medium-sized, and some smaller ones, before the start of the treatment.

### 2.8. Drug Treatment of Zebrafish Embryos Bearing Human Tumor Cells

Three drug doses (MTD, ¼ MTD, 1/10 MTD) were administered for INF_R_1288_r1 and INF_R_1467_r1 and two doses (MTD, ¼ MTD) were administered for INF_R_359_r3. The drugs were administered in 1 × E3 buffer supplemented with 0.2 mM PTU. The treatment of zebrafish embryos started after the first imaging time point at 24 hpi (baseline: start of treatment) for 48h (end of experiment 72 hpi). It is generally estimated that zebrafish embryos take up approximately 1/20 to 1/10 of the drug concentration that has been applied [17,27].

### 2.9. Imaging and Analysis of Zebrafish Embryos Bearing Human Tumor Cells

Imaging of embryos was performed 24 hpi and 72 hpi at 32–34 °C using the ImageXpress Micro Confocal High-Content Microscope (Molecular Devices, San José, CA, USA). Embryos were anesthetized using 1x tricaine and were put into Hashimoto (Funakoshi Co., Ltd., Tokyo, Japan) zebrafish 96-well plates with one embryo/well. Images were taken with 4x magnification in multiple z-stacks (35 z-stacks per image; step size 20 µM) and two sites per well covering the whole yolk sac, using brightfield and Cy3 settings, respectively. The tumor volume was calculated with an automated analysis pipeline (in-house macro for ImageJ), carefully discriminating specific signals from yolk sac-caused blurry background signal, as described previously [26]. To determine the difference (e.g., increase due to growth) of tumor volume from baseline until end of treatment, the percentage of volume difference was calculated between these two imaging time points.

The zebrafish-adapted Response Evaluation Criteria in Solid Tumors (RECIST) as described previously [26] was used to evaluate and visualize the drug response. To be classified as progressive disease (PD) the tumor volume must have increased at least 20%, and to be classified as partial response (PR) the tumor volume must have decreased by more than 30%.

### 2.10. Immunohistochemistry (IHC)

As described previously [26], embryos were fixated in Special Fixative for Anatomy and Histology (Morphisto, Frankfurt am Main, Germany) for 24 h at 4 °C. The fixated zebrafish embryos were placed into embedding cassettes with biopsy pads prior to dehydration by incubation in graded ethanol series (50% ethanol to absolute ethanol) with the Leica ASP300S tissue processor (Leica Biosystems, Wetzlar, Germany), followed by incubation in firstly xylene and secondly paraffin. Next, the embryos were embedded in paraffin, cut into 3 µm sections with a rotary microtome (Microm HM 355 S, Thermo Fisher) and mounted onto glass microscopy slides.

For nuclear staining, the paraffin sections were deparaffinized and rehydrated in a descending alcohol series. After drying, they were washed twice with Tris buffer pH 7.4 and stained 1–2 min with DAPI (1:2000, 10 mg/mL stock solution; Biotium, Fremont, CA, USA). After washing again three times with Tris buffer, they were covered with fluorescence mounting medium (Dako Omnis, Agilent, Santa Clara, CA, USA) and stored at +4 °C. DAPI images were acquired with a Nikon Ts2-FL inverted microscope using a C-LED385 Epi-FL filterblock and CFI P-Fluor DL 10 × Ph1/0.30 and CFI S P-Fluor ELWD ADM 20 × C/0.45 objectives.

Haematoxylin-eosin staining (haemalaun: Carl Roth, Karlsruhe, Germany; eosin: Merck, Darmstadt, Germany) was carried out according to the standard protocol on paraffin sections previously deparaffinized with xylene and then dehydrated in descending alcohol series. Finally, the samples were covered in Eukitt mounting medium. Overview and detailed images of the H&E-stained zebrafish embryos were taken with a Zeiss Cell Observer.Z1 widefield microscope using the tile scanning and stitching function in transmitted light and the following objective lenses: 5×/0.16 EC Plan-NEO Ph1 DIC0, 10×/0.3 Plan-NEO Ph1 DICIII, and 20×/0.8 Plan-Apo DICII.

For SMARCB1 and Ki-67 immunohistochemistry 5-µM-thick formalin-fixed, paraffin-embedded (FFPE) tissue sections were used. These were mounted on StarFrost Advanced Adhesive slides (Engelbrecht, Kassel, Germany) followed by drying at 80 °C for 15 min. Immunohistochemistry was performed on a BenchMark Ultra immunostainer (Ventana Medical Systems, Tucson, AZ, USA). Sections were stained with SMARCB1 and Ki-67. For SMARCB1 staining, slides were deparaffinized and pretreated at 90 °C in Cell Conditioning 1 buffer (Ventana) for 92 min. The sections were incubated with the primary antibody (1:200; INI-1, clone MRQ-27, Cell Marque, USA) for 1 h. For Ki-67 staining, slides were deparaffinized and pretreated at 92 °C in Cell Conditioning 1 buffer (Ventana) for 64 min. The sections were incubated with the primary antibody (1:100; clone MIB-1, DAKO) for 32 min. OptiView DAB Detection Kit (Ventana Medical Systems) was used for visualization.

### 2.11. Reagents for the Treatment of Zebrafish Embryos

Reagents used for the treatment of the zebrafish-xenograft models are listed in Table 2.

### 2.12. Ethical Approval

Experiments involving human patient material were performed in accordance with the Declaration of Helsinki and were approved by the ethics committee of the Medical Faculty University Hospital Heidelberg. All applicable national and institutional guidelines for the care and use of zebrafish were followed. All mouse experiments were conducted in accordance with legal and ethical regulations and approved by the regional council (Regierungspraesidium Karlsruhe, Germany; G-2/20). All procedures performed involving animals were in accordance with national and institutional ethical standards. The INFORM Registry is registered in the German Clinical Trial Register with the following ID: DRKS00007623. Ethics committee approval for INFORM was obtained from Heidelberg University Hospital’s review board. Informed consent was obtained from all subjects involved in the study. 

## 3. Results

Ex vivo functional assays with patient-derived material are promising tools in precision oncology. We used three different culture and propagation methods of patient-derived tumor cells. (1) Patient-derived spheroid cultures shortly after tumor dissociation (fresh tissue culture/FTC). (2) Corresponding long-term tumoroid-like cultures (LTC). Here, patient-derived cells are kept and propagated as three-dimensional matrix-free cultures in stem-cell serum-free medium for at least six passages. (3) Reisolated patient-derived cells from mouse PDX tumors. Here, mPDX tumors are used to propagate patient-derived tumor cells in vivo, and cells are later re-isolated and used for the drug screen. To characterize the three different ex vivo culture models, we used three patient-derived tumor samples from the INFORM study (Table 3) and compared the sensitivity of the tumor-derived cell culture models to 76 clinically relevant anti-cancer drugs.

### 3.1. Characterization and Comparison of the Three Patient-Derived Samples

**Figure 1:**
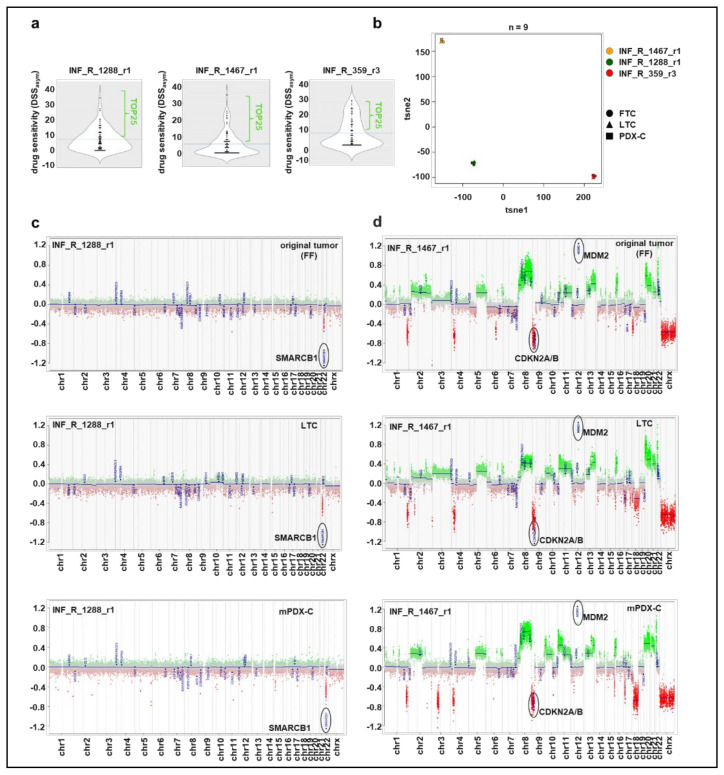
Comparison of the original tumor with matched culture models. (**a**) Overall drug sensitivity (DSS_asym_) against 76 clinically relevant drugs of the three patient-derived samples (FTCs), measured with CellTiterGlo metabolic activity assay. Horizontal line reflects the mean. (**b**) t-SNE analysis of DNA methylation profiles for comparison of the original tumors and their tumor-derived culture models LTC and mPDX-C. (**c**) Copy-number profiles of the original rhabdoid tumor INF_R_1288_r1, and its LTC and mPDX-C models reveal similar genome-wide methylation patterns and recurrent *SMARCB1* deletion, characteristic for rhabdoid tumors. (**d**) Copy-number profiles of the original eRMS tumor INF_R_1467_r1, and its LTC and mPDX-C models reveal similar genome-wide methylation patterns and recurrent high-level *MDM2* amplification, *CDKN2A/B* deletion, LOH and instable genome. FTC: fresh tissue culture; FF: fresh frozen; LTC: long-term culture; mPDX-C: mouse-PDX-derived culture; LOH: loss of heterozygosity.

We generated culture models of tissue samples derived from three different patients enrolled in the INFORM registry, which showed moderate to good drug response in the INFORM ex vivo drug screen profiling [3,4] (and Peterziel et al., manuscript in preparation) (Figure 1a). The tumor-derived cells were kept as three-dimensional spheroid cultures in a serum-free stem cell medium. Drug sensitivity of the FTC samples was tested for at least 76 clinically relevant drugs (mostly approved or late-phase clinical trials). The drug response was evaluated via the drug sensitivity score (DSS) as proposed by Yadav et al. for high-throughput compound testing studies [28] with minor modifications (DSS_asym_) [25]. We first confirmed that the different culture models reflected the molecular profile of the original tumor via DNA methylation analysis. DNA methylation arrays are regularly used for classifying pediatric tumors e.g., through a t-distributed stochastic neighbor embedding (t-SNE) analysis and also allow for determining low profile copy number alterations [29]. The t-SNE analysis clearly grouped the three culture samples of each patient together by the measured methylation patterns (Figure 1b). Moreover, each tumor entity (rhabdoid tumor, eRMS, neuroblastoma) clustered to the respective reference set [23,24] (Figure S1a). Further, copy number variation (CNV) plots proved that characteristic alterations of the original tumor were still present in both spheroidal long-term cultures (LTC) and spheroidal mouse-PDX-derived cultures (mPDX-C) (Figure 1c,d and Figure S1). We conclude that for the here tested three cases, the three-dimensional long-term culture of tumor tissue (either directly patient-derived or propagated in mice and xenograft-derived) in serum-free stem cell medium preserved the molecular characteristics of the original tumor.

### 3.2. Comparison of Drug Sensitivities of Short-Term, Long-Term and Mouse-Xenograft Derived-Cultures Obtained from the Same Original Tumor

**Figure 2:**
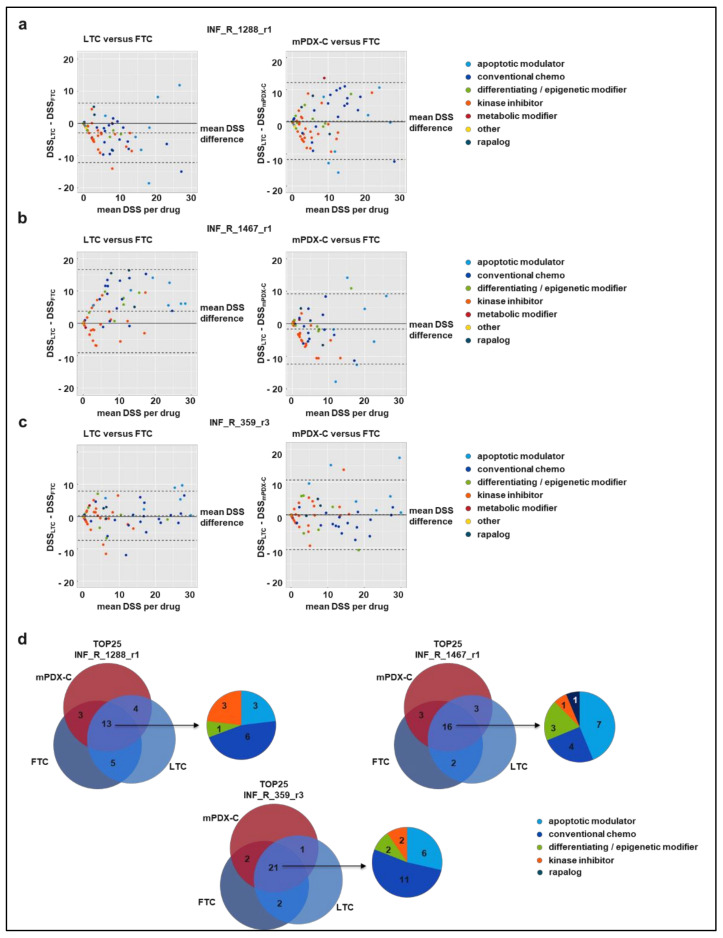
DSP comparison. (**a**–**c**) Bland-Altman plots for comparison of DSS scores of each drug for LTC (left panel, **a**–**c**) or mPDX-C (right panel, **a**–**c**) versus FTC. The difference between the DSS is plotted (*y*-axis) against the average (mean) DSS for each drug. Color code on the right reflects the different drug classes. Cultures were derived from patient sample INF_R_1288_r1 (**a**), INF_R_1467_r1 (**b**) and INF_R_359_r3 (**c**). (**d**) Venn diagrams show the overlap of TOP25 drugs throughout all three culturing methods for each of the samples, as well as the broad classes these drugs belong to. DSS: Drugs Sensitivity Score; LTC: long-term culture; mPDX-C: mouse-PDX-derived culture; FTC: fresh tissue-derived culture. CI: confidence interval.

We used Bland-Altman analysis to test whether the different culture models (FTC, LTC, mPDX-C) showed similar responsiveness to individual drugs (Figure 2a–c). The overall agreement between the models was quite coherent. However, we observed outliers beyond the limits of agreement (LoA) for all tested models (rhabdoid tumor INF_R_1288_r1: *n* = 4–5; eRMS INF_R_1467_r1: *n* = 0–3; neuroblastoma INF_R_359_r3: *n* = 3–5). In general, mPDX-C and FTC showed very good agreement, with no (INF_R_1288_r1; INF_R_359_r3) or marginal (INF_R_1467_r1) shifts in mean drug sensitivity. When comparing FTC and LTC, we noticed a shift in mean drug sensitivity for the rhabdoid tumor INF_R_1288_r1 (FTC > LTC) and the eRMS INF_R_1467_r1 (FTC < LTC) models. In contrast, agreement of FTC and LTC drug response was very good in the case of neuroblastoma INF_R_359_r3, with no shift in mean drug sensitivity and an overall small CI.

The DSS is calculated based on the area under the dose–response curve. As curve fitting is more error-prone for drugs with weak or no effect, DSS values are less accurate for such drugs. We thus ranked DSS_asym_ scores and focused on the TOP25 drug hits for all samples and models. Figure 2d illustrates the overlap of the TOP25 hits in Venn diagrams. For the rhabdoid tumor INF_R_1288_r1, 13 drugs (52 %) are among the TOP25 drug hits for all three culture models and 6/13 (46%) of these overlapping hits are conventional chemotherapy. For the eRMS INF_R_1467_r1, 16 drugs (64%) are among the TOP25 drug hits for all three culture models and 7/16 (44%) of these overlapping hits are apoptotic modulators. For the neuroblastoma INF_R_359_r3, 21 drugs (84%) are among the TOP25 drug hits for all three culture models and 11/21 (52%) of these overlapping hits are conventional chemotherapy.

In summary, the overall drug sensitivity shifted only between the LTC and FTC models for two of the tested tumor samples, with no clear preference towards one model being more sensitive than the other. Analysis of the TOP25 drug hit list revealed high similarity in the detected drug hits (52–84%) between the FTC, LTC and mPDX-C models of all tumor samples.

### 3.3. Drug Hit Selection and Zebrafish Embryo Toxicity Test

To validate the drug screen results in vivo on our zebrafish embryo xenograft model, we selected a subset of TOP25 hit drugs that overlapped in FTC, LTC and mPDX-C models and focused only on those that were effective in clinically achievable concentrations. In addition, we included the major drug hits according to molecular tumor board recommendation for in vivo testing. However, some of these are overlapping with the DSP-derived TOP25. Table 4 lists the selected drugs as well as negative controls, i.e., drugs with no effect in the drug screen profiling (DSP).

**Figure 3:**
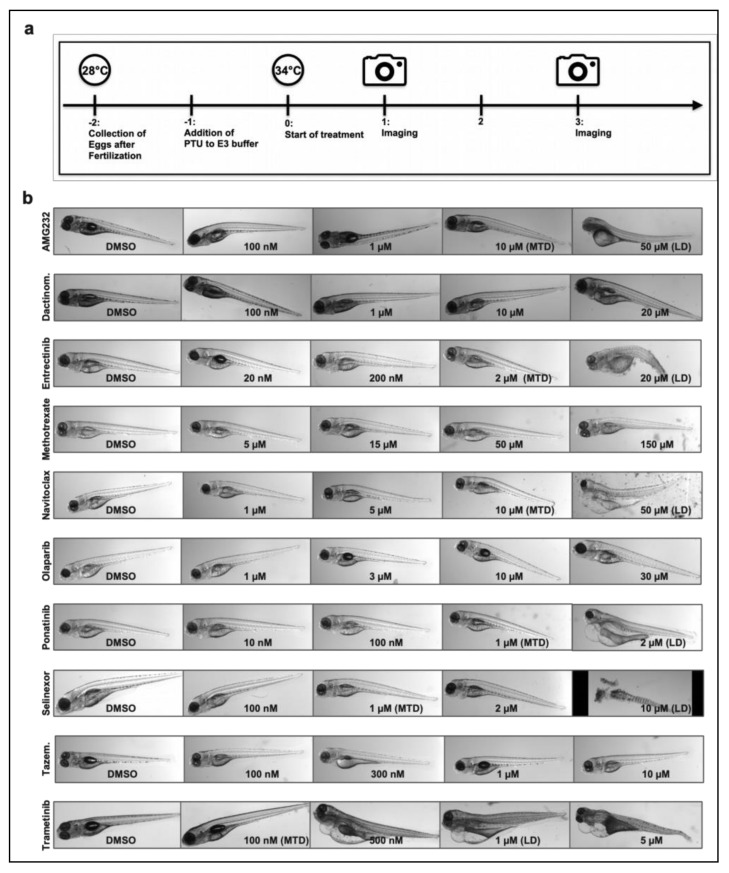
Zebrafish embryo drug toxicity test with selected validation candidates. (**a**) Timeline. Zebrafish embryo started receiving treatment on experimental day 0 (48 hpf) and embryos (*n* = 3 per concentration) were imaged on experimental day one and day three. (**b**) Representative images of embryos treated with drug hits of interest for subsequent validation studies.

When testing drug effects on zebrafish embryos, substances are applied to the surrounding buffer and have to be taken up by the embryos. It is estimated that the extent of compound uptake by zebrafish embryo is 1/10 to 1/20 of the cell culture treatment concentration [16]. Thus, before testing drug efficacy in zPDX, we needed to determine the maximum tolerated dose (MTD, i.e., the dose without harmful effects on zebrafish embryos) for each compound in Table 4. We therefore performed drug toxicity tests on zebrafish embryos for 72h (Figure 3a). With this test, we determined the lethal dose (LD) and maximal tolerated dose (MTD) for each compound used (Table 5; Figure 3b). The MTD is defined as the highest concentration where embryos did not succumb to treatment or showed signs of developmental malformations. In the case of poor drug solubility where LD could not be reached, MTD was defined as the highest achievable concentration without noticeable drug precipitation.

In line with the above-named studies, the determined MTDs for the drugs of interest were up to 20-fold higher than the concentrations commonly used for cell culture studies.

### 3.4. Drug Hit Validation with zPDX

**Figure 4:**
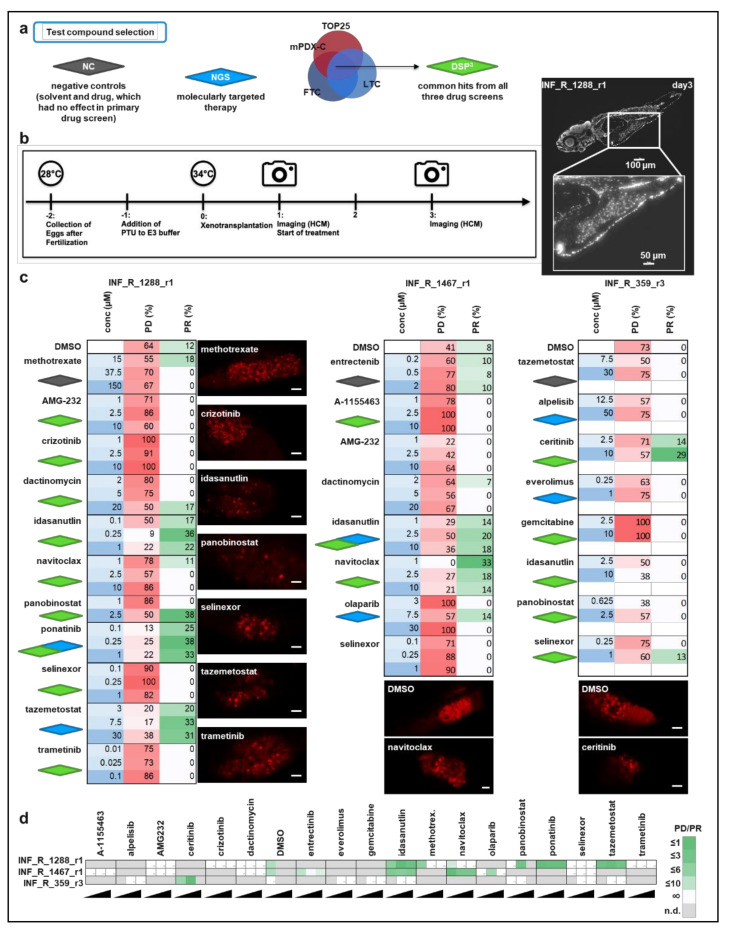
In vivo drug hit validation with the zPDX tumor growth model. (**a**) Scheme of hit selection for validation experiments. (**b**) Scheme and timeline of experimental setup. Fluorescently labeled tumor cells were injected into the yolk sac on experimental day zero and from that time point on, the embryos were kept at 34 °C. The treatment started on experimental day one. Imaging of the embryos was performed on experimental day one before treatment and on day three (the end of the experiment). Microscopic images on the right of the timeline display an untreated day three tumor at two magnifications (scale bar 100 µM; inlay: scale bar 50 µM). Nuclei were stained with DAPI to visualize the tumor cells in the yolk sac. (**c**) Effects of drug of interest on tumor growth in the zPDX model. Tumor progression was monitored by automated quantification of tumor volume on day one and day three according to Response Evaluation Criteria in Solid Tumors (RECIST) 1.1 adopted for zebrafish tumors. Numbers indicate the percentage of early larvae with PD (red color) or PR (green color) in each treatment group on day three. Buffer-applied concentrations in µM are marked in blue. Grey diamonds: negative control treatments; green: DSP common hit in all three culture models; blue NGS suggested hit. Number of embryos per treatment group for INF_R_1288_r1 (*n* = 4–42, pooled from two experiments), INF_R_1467_r1 (*n* = 5–39, pooled from two experiments) and INF-R-359_r3 (*n* = 7–8, from one experiment). Images aside or below the heatmap display DiI-stained tumor cells of the respective model with selected treatments on day three. Scale bar: 50 µM. (**d**) Heatmap reflecting the ratio of PD to PR (green shading) for all treatments and all models (*n* = 4–42). Grey: not detected (n.d.); ∞: the percentage of PR was 0%. PD: progressive disease, tumor volume must have increased at least 20%; PR: partial response, tumor volume must have decreased by more than 30%.

As the selected drug hits (Table 4) were shared amongst FTC, LTC and mPDX-C models (DSP^3^) and the difference in responsiveness to these drugs between the culture models were within a reasonable range, we used the LTC model for in vivo drug hit validation in our zPDX model. Zebrafish embryos were xenotransplanted 48h post-fertilization with LTC cells of rhabdoid tumor INF_R_1288_r1, eRMS INF_R_1467_r1 and neuroblastoma INF_R_359_r3, respectively and transferred to 96-well plates. Drugs of interest were added 24h post-injection (day one) at the MTD, ½ MTD and ¼ MTD, respectively. Solvent and selected drugs without effect in the DSP were used as negative controls (Table 4, Figure 4a). Early fish larvae were imaged via high-content microscopy on day one and day three, respectively, and drug effects were calculated based on the change in tumor volume from day one to day three (Figure 4b). Changes in tumor volume were expressed as a percentage of progressive disease (PD) and percentage of partial response (PR) according to RECIST adopted for zebrafish xenografts [26]. The result for each patient sample is summarized in a heatmap depicting the applied concentrations, % PD and % PR. DSP^3^ hits are marked with the green, negative controls with the gray and NGS-proposed drugs with the blue diamond symbol (Figure 4c). Representative microscopic images of day three tumors are depicted for some drugs aside or below the respective heatmap.

From all 10 tested treatments, the INF_R_1288_r1 rhabdoid tumor model harboring a *SMARCB1* deletion responded best to the treatment with p53-MDM2 inhibitor idasanutlin (DSP^3^ hit), RTK (receptor tyrosine kinase) inhibitor ponatinib (DSP^3^ and NGS hit) and EZH inhibitor tazemetostat (NGS hit), as these treatments substantially decreased the number of tumors with progress (PD) and improved the partial response rate. Engraftment and mitosis of SMARCB1 negative tumor cells on day three is exemplarily shown in Figure S2a–c, with immunostaining against SMARCB1 and Ki-67. The embryonal RMS INF_R_1467_r1 model displayed an improved response rate when treated with the two apoptotic modulators idasanutlin (DSP^3^ and NGS hit) and BCL2 family inhibitor navitoclax (DSP^3^ hit). Only one drug, ALK inhibitor ceritinib (DSP^3^ hit), substantially decreased the tumor volume in 30% of the INF_R_359_r3 neuroblastoma zPDX models (Figure 4c). Two of the effective drugs (ponatinib for INF_R_1288_r1 and idasanutlin for INF_R_1467_r1) showed an overlap with the NGS proposed therapy. One NGS proposed drug, tazemetostat, was effective in the zPDXmodel, although it was not within the DSP^3^ hit list. To compare the drug effect of all tested drugs in all three zPDX models, we calculated the ratio of %PD divided by %PR and displayed it in a heatmap (Figure 4d). A ratio below 1 means that more tumors of the model regress than progress after treatment. Higher ratio numbers are reflected in lighter colors and reflect more progress than regression. White color indicates a non-calculable ratio, as the % of PR was 0. Gray color indicates that the drug effect was not determined for this model. 

**Figure 5:**
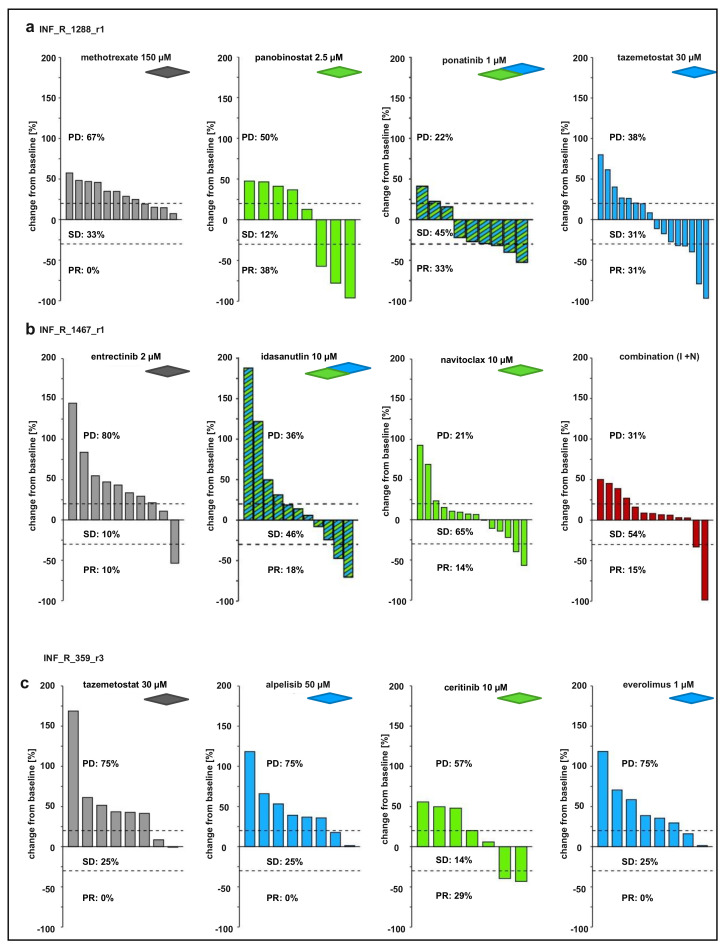
Waterfall plots demonstrating change in tumor volume for (**a**) the INF_R_1288_r1 rhabdoid tumor zPDX model (methotrexate with 12 individual embryos and ponatinib with 9 individual embryos, each from one experiment; panobinostat with eight individual embryos and tazemetostat with 16 individual embryos, both from two pooled experiments), (**b**) the INF_R_1467_r1 eRMS zPDX model (idasanutlin with 11 individual embryos from one experiment; entrectinib with ten individual embryos and navitoclax with 14 individual embryos, both from two pooled experiments), and (**c**) the INF_R_359_r3 neuroblastoma zPDX model (tazemetostat with eight individual embryos, alpelisib with eight individual embryos, ceritinib with seven individual embryos and everolimus with eight individual embryos, all from one experiment). Depicted is the change in tumor volume (%) for each individual zebrafish early larvae engrafted with tumor cells, from baseline (day one = start of the treatment) to day three after tumor implantation. Numbers indicate the percentage of early larvae with progressive disease (PD), stable disease (SD) and partial response (PR) in each treatment group on day three.

Data for the highest concentration of each drug were also expressed as waterfall plots comparing the change in tumor volume over baseline of individual fish embryos (Figure 5 and Figure S3–5). In addition to the above-mentioned drug hits, the response rate of INF_R_1288_r1-derived tumors also improved upon the treatment with HDAC inhibitor panobinostat (3 of 8 tumors show at least a 30% decrease in tumor volume) (Figure 5a). The combination of panobinostat with tazemetostat slightly improved the treatment response, reflected in the shifting of the PD-to-PR ratio from above 1, meaning more progress than response, down to 1.0 (Figure S3). For INF_R_1467_r1-derived tumors, we further tested the combination of the above-identified hit compounds, idasanutlin and navitoclax. However, 2 of 13 tumors showed partial response, which is similar to a single compound treatment, meaning that the combination of both drugs did not improve the response rate (Figure 5b; Figure S4). As described above, only the ALK inhibitor ceritinib, improved the response rate (2 of 7) of the neuroblastoma INF_R_359_r3 zPDX model (Figure 5c; Figure S5). The combination of ceritinib with alpelisib (PI3Ki) did not further increase this effect (Figure S5). Together, these results show that the zPDX-model generated with the patient-derived LTC is suitable to narrow down the hit list and to test drug combinations.

## 4. Discussion

Personalized medicine in childhood cancer aims for the identification of actionable alterations and some promising molecular drug targets are already being translated into clinical applications (e.g., ALK, NTRK, MET, BRAF) [4]. To identify more therapeutic vulnerabilities, especially for tumors without a clear actionable driver alteration, personalized preclinical functional models are employed. These serve as avatars to facilitate a drug sensitivity profiling for the identification of tumor-targeting, individualized, treatment strategies. Commonly used models for adult tumors comprise patient-derived (orthotopic) mouse xenografts (mPDXs) and matrix-based organoid cultures, which both have their limitations for pediatric and embryonal tumors. The mPDX models are cost-intensive, and not all pediatric tumors easily engraft [20]. Matrix-based organoid cultures work best for epithelial cells and are quite useful for adult carcinomas but are less applicable for embryonic (neuro)blastomas or sarcomas [30]. However, embryonic tumor cultures often spontaneously form spheroid aggregates without the need for a supportive matrix [30]. Tissue-derived tumor spheres are hence arising as attractive models for ex vivo functional precision medicine studies of pediatric or embryonal tumors. The freshly isolated primary cells reflect the genetic and the clonal heterogeneity of the native tumor generally quite well, containing different cell types and thus enhancing the predictiveness of the pre-clinical model. These tumoroids are kept in an organoid-like culture under serum-free conditions in a stem-cell medium supplemented with growth factors. Such non-adherent models enable more cell–cell interactions and reflect the metabolic heterogeneity of a tumor with areas of maximal growth, slow-growing starved regions or even necrotic core regions [6,31]. Here we first compared the drug sensitivity profile towards 76 clinically relevant anti-cancer drugs of three culture models to targeted and standard-of-care therapy and then decided on one culture model, the LTC, for zPDX add-on experiments.

Ex vivo drug sensitivity assays are emerging in pediatric and adult functional precision oncology as an approach to identify candidate drugs for the anti-cancer treatment of particularly relapsed and high-risk patients [28,32,33,34,35,36]. We used the profiling for the characterization of the three ex vivo culture models: (i) patient-derived spheroid cultures shortly after tumor dissociation (FTC); (ii) corresponding long-term organoid-like cultures (LTC) kept under serum-free conditions in stem-cell medium supplemented with growth factors; (iii) reisolated patient-derived cells propagated in mouse PDX tumors shortly after tumor dissociation (mPDX-C). We found a shift in overall drug sensitivity between two of three LTC and FTC models, but no shift between mPDX-cultures and FTC models. When comparing LTC and FTC, there was no clear preference towards the one or the other model being more sensitive in terms of mean drug sensitivity than the other is. The differences between the culture models diminished when we compared the TOP25 hit list, which revealed an overlap of at least 50% of the drugs between all three culture models. FTC and mPDX-C have the benefit of cell-type heterogeneity, as the whole sample is dissociated without tumor cell purification and outgrowth. The limitation is the critical low amount of material available from the tumor resection. The LTC model may have lost slowly and non-proliferative cells or sub-clones over time but yields enough material for several functional assays. The mPDX model is severely limited by its time frame. mPDX models grow over the course of several weeks or months, which might be too slow in a clinical setting. Still, mPDX models are very useful in propagating patient-derived tumor cells that are unable to proliferate in cell culture in the first place, whereas the reisolated cells of the mPDX often successfully establish a long-term organoid-like culture, very likely due to the higher starting cell number. Despite all these differences, the CNV plots and 850k methylation array analysis revealed high similarity in the copy-number variation profile and clustering to the respective original tumor and tumor entity in the tSNE plot. Of course, these methods might overlook phenotype changes based on altered expression and activity levels of single genes or enzymes. Nevertheless, for our three models, the three-dimensional cultures of tumor tissue in serum-free stem cell medium, either as LTC or as mPDX-C, preserved the main molecular characteristics of the original tumor.

Zebrafish embryos without tumor cell implantation are used for toxicity assays, also known as a fish embryotoxicity test (FET) [37,38]. We have used an adopted version (at 34 °C instead of 28 °C) of the FET to determine the MTD and LD of our drug library and presented the data for the drugs used in our zPDX experiments. In fact, the assay allows for the assessment of toxicity with all varieties of samples of interest in a high-throughput manner, as here the embryos are used without tumor cell injections [38]. Hence, the assay has emerged as the leading-edge method of toxicology research for testing drugs and particularly so far unknown drug combinations for the potential use in patients due to the short time required for analyses, transparency of embryos, short life cycle, high fertility, and genetic data similarity [38].

Patient-derived (orthotopic) mouse xenografts (mPDXs) are largely accepted as patient avatars to test drug sensitivities. As these models are not always successful in tumor cell engraftment and typically take too much time to be directly considered for the patient’s treatment plan, the zebrafish (Danio rerio) embryo PDX model (zPDX) provides a faster alternative xenograft model in the field of functional precision medicine [39,40]. We applied the zPDX model as an add-on to functional drug screens after hit finding for hit validation and combination testing. As the differences amongst FTC, LTC, and mPDX-C models were within a reasonable range, we decided to use the more feasible LTCs. In future studies, however, we will focus on the establishment of fresh tissue zPDX and testing of a condensed zPDX optimized drug library for rapid in vivo to clinic translation. LTCs have the benefit of being molecularly characterized and yielding enough viable cells to compare several drugs and drug combinations in parallel with appropriate group sizes but have the disadvantage that the establishment of the fully characterized and stable culture (>6 passages) takes weeks to months, which might be too long to stick to the clinical timeframe.

The first sample we employed in our zebrafish embryo model was a rhabdoid tumor. These are pediatric tumors with very poor prognosis and are characterized by SMARCB1 inactivation. SMARCB1 is a member of the SWI/SNF chromatin-remodeling complex and functions as a tumor suppressor in most rhabdoid tumors. The SWI/SNF complex controls the histone methyltransferase complex PRC2. Loss of SMARCB1 impairs SWI/SNF’s function, leading to deregulated PRC2 activity, particularly of the catalytic subunit, EZH2, which is driving tumor progression [41,42,43]. The FDA-approved EZH2 inhibitor tazemetostat interferes with this mechanism and stops tumor growth [44]. In our study, the zebrafish embryo model of the rhabdoid tumor sample harboring the typical *SMARCB1* deletion and a *PRKCD* (protein kinase C delta) mutation responded best to the EZH2 inhibitor tazemetostat and the multi-kinase inhibitor ponatinib out of 11 tested drug hits. Tazemetostat has already been described to be potentially active against rhabdoid tumors [42]. Ponatinib is an FDA and EMA-approved Bcr-Abl inhibitor, which has activity on many kinases, including PDGFRs and FGFRs [45]. Broad inhibitors of tyrosine kinase receptors (RTKs), such as ponatinib, have been identified as SMARCB1-related therapies and particularly PDGFR and FGFR are expressed in rhabdoid tumors upon SMARCB1 loss [46,47]. In addition to EZH2 histone methyltransferase inhibition, targeting of histone deacetylation has also demonstrated anti-tumor activity for rhabdoid tumors in preclinical studies [48,49]. Of note, the HDACi panobinostat was also effective in its highest concentration in our zPDX model, suggesting epigenetic targeted drugs as a potentially supportive therapy here.

The second sample we employed in our zebrafish embryo model was an eRMS with the *MDM2* amplification and so-called BRCAness, which, out of seven drugs tested, responded only to the MDM2 inhibitor idasanutlin, and the BCL2 family inhibitor and apoptosis-inducer navitoclax, which are both currently in clinical development. MDM2 is the negative regulator of the tumor suppressor TP53, leading to its ubiquitination and degradation. *MDM2* amplification has been detected in several human adult and pediatric tumors, including pediatric rhabdomyosarcomas [50,51]. MDM2 overexpression impairs the effect of chemotherapy and MDM2 inhibitors, which block the binding of MDM2 and TP53, stabilize TP53 levels and activate the TP53 signaling pathway, resulting in cell cycle arrest and induction of apoptosis [50]. Surprisingly, the combination of both drugs did not further improve the response rate in our model.

The third sample, the zPDX from the neuroblastoma culture with *MYCN* amplification and *PIK3CA* (phosphatidylinositol-4,5-bisphosphate 3-kinase catalytic subunit alpha) mutation, responded only moderately to the ALK inhibitor ceritinib out of five tested drugs. In neuroblastomas, abnormal receptor tyrosine kinase (RTK) activity (e.g., ALK, NTRK) occurs frequently, but the frequency of genetic aberrations of downstream effectors, such as PIK3CA is rather low [52]. The surprising finding of the ALK inhibitor ceritinib being (moderately) effective, without detecting *ALK* aberrations by NGS might be explained by the fact that also wild-type *ALK* can act in an oncogenic manner as long as its expression is above a certain threshold [53]. There are several activated pathways downstream of ALK, including MAPK-ERK and PI3K/Akt/mTOR. As this tumor sample displayed a *PIK3CA* mutation, this might explain the overall only moderate response to ceritinib, despite being the only sensitivity detected in the zPDX model. The promising results of these three cases point towards the opportunity to use this model in the future in a broader approach testing several tumors with similar genetic backgrounds for their drugs response patterns e.g., to identify novel genetic biomarkers predicting certain drug/drug class sensitivities. This will be of particular interest for undruggable molecular alterations such as *MYC* or transcription factor fusion-positive tumors.

The first drug testing experiment using zPDX was reported more than 15 years ago [54] and the model has now consolidated in the field of precision oncology [55,56,57]. In a retrospective study of colorectal patients, the zebrafish avatars were predictive for the clinical outcome in four out of five patients [27]. A recent study showed that the use of the zebrafish embryos as avatars for patients affected by pancreatic ductal adenocarcinoma has the potential to be predictive for the chemotherapy response of individual patients [40]. This study very elegantly used small pieces of stained tissue and transplanted these pieces into the yolk of zebrafish embryos [40,58]. The model was predictive if compared to the response rates of the tumor to the different chemotherapy protocols known from the literature and was further validated in a prospective study with PDAC patients [59]. From 16 zPDX models, the chemosensitivity profile was compared to the clinical response to adjuvant chemotherapy. The model predicted relapse/no relapse within one year after surgery for 12 out of 16 patients and was able to identify those patients who responded to chemotherapy [59]. A co-clinical trial (ClinicalTrials.gov Identifier:NCT00070213) is currently running to test the zPDX model with more solid cancers [58].

Despite this success, the zebrafish embryo model also has some pitfalls, such as the short incubation time, the incubation temperature and the immature immune system. Most studies use a 48h incubation time for the drug sensitivity testing, which works very well for cell death inducers, but might be too short to detect drugs acting through cell cycle interference or epigenetic mechanisms, which usually need a longer time to take effect. Another limitation of the zebrafish xenograft model is the relatively high variation of tumor growth within the treatment groups. The main cause for this lies within the technique of microinjection of cells, which tend to clump after a certain time, leading to experimental changes (cutting the tip of the needle or increasing the pressure or exchange of the needle) preventing injection of all embryos with the exact same parameters. Most of the human cancer xenograft studies in zebrafish embryos have used incubation temperatures in the range of 32–34 °C. When using 34 °C, human cancer cells still proliferate, though with a slightly increased population doubling time [60] (and own observations), which again might hamper the detection of drugs acting through cell cycle interference, leading to an underestimation of drug effects. As targeted therapy often uses enzyme inhibitors for the blockade of overactive kinases (e.g., ALK, MAPK, NTRK), the question arises, whether enzyme activity per se is reduced at 34 °C, as the optimum temperature for enzymes located in human cells is around 37 °C. However, some studies point in the direction that human enzymes are still similarly active at 34 °C [61,62,63]. Whether the human enzyme activity is significantly impaired at 34 °C affecting the results of drug sensitivity profiling including kinase inhibitors will be addressed in future studies. One recent study suggests increasing the temperature to 36 °C for the first 24 to 48h after tumor cell transplantation [64], an option we plan to test in our models in the future. Just as in immunocompromised mice, the immature adaptive immune system in *danio rerio* embryos enables xenotransplantation of human material without immunosuppression. However, it also prevents studying interactions of tumor cells and the host immune system [65]. It is possible, however, to evaluate CAR (chimeric antigen receptor) T cell-mediated killing of human cancer cells in zebra fish, as shown by applying CD19-specific CARs versus Nalm-6 leukemia cells [66].

## 5. Conclusions

Taken together, the zPDX might be particularly useful when investigating drugs that induce programmed cell death and do not depend on cellular proliferation, and as shown by our study with three samples, to rank and narrow down the hit list to the most effective drugs and drug combinations. This includes the selection and reduction of agents for preclinical testing in mice and provides in total a comprehensive data set to inform clinical studies. With this, the model has the potential to give reasonable additional information and value for personalized precision medicine. Our future studies will focus on the establishment of fresh tissue zPDX and the comparison of drug responses of the fresh tissue zPDX model to the LTC zPDX model.

## Figures and Tables

**Table 1:** Cell numbers and pre-culture time/passage number at drug screen.

Culture	Cells/Well	Pre-Culture Time ^1^/Passage
FTC_INF_R_1288_r1	908	5 d after fresh tissue dissociation
LTC_INF_R_1288_r1	1000	passage 22
PDX-C_INF_R_1288_r1	1000	2d after PDX tumor dissociation
FTC_INF_R_1467_r1	1000	3 d after fresh tissue dissociation
LTC_INF_R_1467_r1	1000	passage 15
PDX-C_INF_R_1467_r1	1000	6 days after PDX tumor dissociation
FTC_ INF_R_359_r3	1000	3 d after fresh tissue dissociation
LTC_ INF_R_359_r3	1000	passage 10
PDX-C_ INF_R_359_r3	1000	14 d after PDX tumor dissociation

^1^ before performing the drug screen

**Table 2:** Reagents used for the treatment of the zebrafish-xenograft models.

Reagent	Stock Concentration/Solvent	Supplier (Name, City, State)
A-1151852	100 mM/DMSO	ChemieTek, Indianapolis, IN, USA
alpelisib	10 mM/DMSO	MedChemExpress, Monmouth Junction, NJ, USA
AMG-232	10 mM/DMSO	MedChemExpress, Monmouth Junction, NJ, USA
crizotinib	50 mM/DMSO	Selleckchem, Houston, TX, USA
dactinomycin	10 mM/DMSO	MedChemExpress, Monmouth Junction, NJ, USA
entrectinib	10 mM/DMSO	MedChemExpress, Monmouth Junction, NJ, USA
everolimus	10 mM/DMSO	LC Laboratories, Woburn, MA, USA
gemcitabine	10 mM/DMSO	MedChemExpress, Monmouth Junction, NJ, USA
idasanutlin	100 mM/DMSO	MedChemExpress, Monmouth Junction, NJ, USA
methotrexate	50 mM/DMSO	Sigma-Aldrich Chemie, Taufkirchen, Germany
navitoclax	100 mM/DMSO	MedChemExpress, Monmouth Junction, NJ, USA
olaparib	100 mM/DMSO	LC Laboratories, Woburn, MA, USA
panobinostat	10 mM/DMSO	LC Laboratories, Woburn, MA, USA
ponatinib	10 mM/DMSO	Selleckchem, Houston, TX, USA
selinexor	100 mM/DMSO	ChemieTek, Indianapolis, IN, USA
tazemetostat	100 mM/DMSO	ChemieTek, Indianapolis, IN, USA
trametinib	25 mM/DMSO	ChemieTek, Indianapolis, IN, USA

**Table 3:** Overview on patient-derived samples.

Patient Sample	Entity ^1^	Molecular Aberrations ^2^
INF_R_1288_r1	rhabdoid tumor	*SMARCB1* del; *PRKCD*mut; *DDR2*mut
INF_R_1467_r1	embryonal RMS (eRMS)	*MDM2*amp; *CDKN2A*mut&LOH
INF_R_359_r3	neuroblastoma	*MYCN*amp; *PIK3CA*mut

^1^ as determined by INFORM methylation profiling; ^2^ as determined by INFORM Next Generation Sequencing (mutations through whole-exome sequencing, copy number alterations through low coverage whole genome sequencing); RMS: rhabdomyosarcoma; del: deletion; mut: mutation; amp: amplification; LOH: loss of heterozygosity; r: relapse.

**Table 4:** Drug selection for zPDX validation.

Basis for Drug Selection	INF_R_1288_r1 (Rhabdoid Tumor)	INF_R_1467_r1 (Embryonal RMS)	INF_R_359_r3 (Neuroblastoma)
NGS based molecular targets ^1^ and matching drugs	tazemetostat (EZH2i; SMARCB1del); ponatinib (RTKi; DDR2mut)	idasanutlin (MDM2i; MDM2amp); olaparib (PARPi; genomic instability)	alpelisib (PI3Ki; PIK3CAmut); everolimus (mTORi; PIK3CAmut)
DSP-derived hits (TOP25 hit for all three models)	AMG-232 crizotinib dactinomycin idasanutlin navitoclax panobinostat ponatinib selinexor trametinib	A-1151852 AMG-232 dactinomycin idasanutlin navitoclax olaparib selinexor	ceritinib gemcitabine idasanutlin selinexor panobinostat
Negative controls (solvent and DSP noneffective drug)	DMSO methotrexate	DMSO entrectinib	DMSO tazemetostat

^1^ as determined by INFORM Next Generation Sequencing (NGS); DSP: drug screen profiling; del: deletion; mut: mutation; amp: amplification.

**Table 5:** Zebrafish Embryo Toxicity Test.

Drug	Concentration Range	MTD ^1^	LD ^2^
A-1151852	1–500 µM	10 µM	not detected
alpelisib	0.1–50 µM	50 µM	not detected
AMG-232	0.1–50 µM	10 µM	50 µM
crizotinib	0.05–10 µM	10 µM	not detected
dactinomycin	0.001–20 µM	20 µM	not detected
entrectinib	0.02–20 µM	2 µM	not detected
everolimus	0.04–10 µM	1 µM	4 µM
gemcitabine	0.1–10 µM	10 µM	not detected
idasanutlin	0.01–50 µM	10 µM	50 µM
methotrexate	1.5–150 µM	150 µM	not detected
navitoclax	1–50 µM	10 µM	50 µM ^3^
olaparib	0.1–30 µM	30 µM	not detected
panobinostat	0.1–20 µM	1 µM	10 µM ^4^
ponatinib	0.01–2 µM	1 µM	2 µM ^5^
selinexor	0.1–20 µM	1 µM	10 µM
tazemetostat	0.1–30 µM	30 µM	not detected
trametinib	0.1–50 µM	0.1 µM	1 µM ^6^

^1^ MTD: maximum tolerated dose; the highest concentration that could be used without observable morbidity, changes in morphology, or behavior; ^2^ LD: lethal dose including the dose leading to signs of toxicity. ^3^ alive, but edema and morphology changes ^4^ 10 µM: alive, but changed heart morphology; 20 µM: dead. ^5^ alive, but edema and morphology changes. ^6^ one embryo dead, two with morphology changes and edema.

## Data Availability

Data is available upon request.

## Supplementary Materials

The following supporting information can be downloaded at: https://www.mdpi.com/article/10.3390/cancers14030849/s1. Figure S1: Comparison of the original tumor with matched culture models, Figure S2: Representative images of immunohistochemistry (IHC) staining, Figure S3: Waterfall plots demonstrating change in tumor volume for the INF_R_1288_r1 rhabdoid tumor zPDX model, Figure S4: Waterfall plots demonstrating change in tumor volume for the INF_R_1467_r1 eRMS zPDX model, Figure S5. Waterfall plots demonstrating change in tumor volume for the INF_R_359_r3 neuroblastoma zPDX model, Table S1: TOP25 Drug Hits for INF_R_1288_r1, Table S2: TOP25 Drug Hits for INF_R_1467_r1, Table S3: TOP25 Drug Hits for INF_R_359_r3.
