# Supplementary material for: Functional Therapeutic Target Validation Using Pediatric Zebrafish Xenograft Models
Source: Cancers (Basel). 2022 Feb 8;14(3):849. doi: 10.3390/cancers14030849 (PMC8834194; doi:10.3390/cancers14030849)
Supplement: Supplementary file 1 [file cancers-14-00849-s001.zip › Supplementary Information_revised_2_corrected.pdf]

# Supplementary Materials:

# Functional Therapeutic Target Validation Using Pediatric Zebrafish Xenograft Models

**Charlotte Gatzweiler [1,2,3], Johannes Ridinger [1,2], Sonja Herter [1,2,4], Xenia F. Gerloff [1,2,4], Dina ElHarouni [1,4,9,10], Yannick Berker [1,2], Roland Imle [4,11,17], Lukas Schmitt [11], Sina Kreth [12], Sabine Stainczyk [12], Simay Ayhan [1,2,4,5], Sara Najafi [1,2,5], Damir Krunic [6], Karen Frese [7], Benjamin Meder [7,8], David Reuss[13], Petra Fiesel [1,14], Kathrin Schramm [1,15,16], Mirjam Blattner-Johnson [1,15,16], David T.W. Jones [1,15,16], Ana Banito [1,11], Frank Westermann [1,12], Sina Oppermann [1,2], Till Milde [1,2,5], Heike Peterziel [1,2], Olaf Witt [1,2,5] and Ina Oehme [1,2*]**

[1] Hopp Children's Cancer Center Heidelberg (KiTZ), 69120 Heidelberg, Germany
[2] Clinical Cooperation Unit Pediatric Oncology, German Cancer Research Center (DKFZ) and German Cancer Consortium (DKTK),, 69120 Heidelberg, Germany
[3] Faculty of Medicine, Heidelberg University, 69120 Heidelberg, Germany
[4] Faculty of Biosciences, Heidelberg University, 69120 Heidelberg, Germany
[5] Department of Pediatric Oncology, Hematology and Immunology, Heidelberg University Hospital, 69120 Heidelberg, Germany
[6] Light Microscopy Facility, German Cancer Research Center (DKFZ), 69120 Heidelberg, Germany
[7] Institute for Cardiomyopathies Heidelberg, Heidelberg University, 69120 Heidelberg, Germany; karen.frese@med.uni-heidelberg.de (K.F.); Benjamin.Meder@med.uni-heidelberg.de (B.M.)
[8] Genome Technology Center, Stanford University, Stanford, CA 94304, USA
[9] Bioinformatics and Omics Data Analytics, German Cancer Research Center (DKFZ), 69120 Heidelberg, Germany
[10] Division of Pediatric Neurooncology, German Cancer Research Center (DKFZ) and German Cancer Consortium (DKTK), 69120 Heidelberg, Germany.
[11] Pediatric Soft Tissue Sarcoma Research Group, German Cancer Research Center (DKFZ), 69120 Heidelberg, Germany
[12] Division of Neuroblastoma Genomics, German Cancer Research Center (DKFZ), 69120 Heidelberg, Germany
[13] Department Neuropathology at Institute of Pathology, Heidelberg University Hospital, 69120 Heidelberg, Germany
[14] Clinical Cooperation Unit Neuropathology, German Cancer Research Center (DKFZ), 69120 Heidelberg, Germany
[15] National Center for Tumor Diseases (NCT) Network, 69120 Heidelberg, Germany
[16] Division of Pediatric Glioma Research, German Cancer Research Center (DKFZ) and German Cancer Consortium (DKTK), 69120 Heidelberg, Germany
[17] Division of Pediatric Surgery, Department of General, Visceral and Transplantation Surgery, University Hospital Heidelberg, 69120 Heidelberg, Germany
* Correspondence: i.oehme@kitz-heidelberg.de

All drugs detected in at least one of the culture models of the rhabdoid tumor sample INF_R_1288_r1 as a TOP25 hit are listed in **Table S1.** For each model it is noted, whether the drug was within the TOP25 hits (yes) or not (no). For the INF_R_1288_r1-derived models, 13 drugs overlapped in all three models.

**Table S1. TOP25 Drug Hits for INF_R_1288_r1**

| Drug name | TOP25 FTC | TOP25 LTC | TOP25 mPDX-C |
|---|---|---|---|
| AMG-232 | yes | yes | yes |
| bortezomib | yes | yes | yes |
| cytarabine | yes | yes | yes |
| dactinomycin | yes | yes | yes |
| daunorubicin | yes | yes | yes |
| idasanutlin | yes | yes | yes |
| panobinostat | yes | yes | yes |
| ponatinib | yes | yes | yes |
| selinexor | yes | yes | yes |
| staurosporine_drug[1] | yes | yes | yes |
| topotecan | yes | yes | yes |
| trametinib | yes | yes | yes |
| vinorelbine | yes | yes | yes |
| A-1155463 | yes | yes | no |

| | | | |
|---|---|---|---|
| A-1210477 | yes | yes | no |
| ceritinib | yes | yes | no |
| entinostat | yes | yes | no |
| navitoclax | yes | yes | no |
| A-1331852 | no | yes | yes |
| doxorubicin | no | yes | yes |
| gemcitabine | no | yes | yes |
| mitoxantrone | no | yes | yes |
| crizotinib | yes | no | yes |
| vinblastine | yes | no | yes |
| vincristine | yes | no | yes |
| cisplatin | yes | no | no |
| sunitinib | yes | no | no |
| temsirolimus | yes | no | no |
| vorinostat | yes | no | no |
| cobimetinib | no | yes | no |
| everolimus | 1no | yes | no |
| thioguanine | no | yes | no |
| axitinib | no | no | yes |
| etoposide | no | no | yes |
| methotrexate | no | no | yes |
| paclitaxel | no | no | yes |
| rapamycin (sirolimus) | no | no | yes |

[1] assay control

All drugs detected in at least one of the culture models of the eRMS tumor sample INF_R_1467_r1 as a TOP25 hit are listed in **Table S2.** For each model it is noted, whether the drug was within the TOP25 hits (yes) or not (no). For the INF_R_1467_r1-derived models, 16 drugs overlapped in all three models.

**Table S2. TOP25 Drug Hits for INF_R_1467_r1**

| Drug name | TOP25 FTC | TOP25 LTC | TOP25 mPDX-C |
|---|---|---|---|
| A-1155463 | yes | yes | yes |
| A-1210477 | yes | yes | yes |
| A-1331852 | yes | yes | yes |
| AMG-232 | yes | yes | yes |
| bortezomib | yes | yes | yes |
| dactinomycin | yes | yes | yes |
| everolimus | yes | yes | yes |
| I-BET151 | yes | yes | yes |
| idasanutlin | yes | yes | yes |
| navitoclax | yes | yes | yes |
| panobinostat | yes | yes | yes |
| selinexor | yes | yes | yes |
| staurosporine_drug [1] | yes | yes | yes |
| topotecan | yes | yes | yes |
| vincristine | yes | yes | yes |
| vorinostat | yes | yes | yes |
| cobimetinib | yes | yes | no |
| trametinib | yes | yes | no |
| rapamycin (sirolimus) | no | yes | yes |
| temsirolimus | no | yes | yes |
| vinorelbine | no | yes | yes |
| crizotinib | yes | no | yes |
| daunorubicin | yes | no | yes |
| entinostat | yes | no | yes |
| doxorubicin | yes | no | no |

| | | | |
|---|---|---|---|
| imatinib | yes | no | no |
| ponatinib | yes | no | no |
| thioguanine | yes | no | no |
| cytarabine | no | yes | no |
| gemcitabine | no | yes | no |
| mitoxantrone | no | yes | no |
| paclitaxel | no | yes | no |
| ceritinib | no | no | yes |
| vinblastine | no | no | yes |
| volasertib | no | no | yes |

[1] assay control

All drugs detected in at least one of the culture models of the neuroblastoma tumor sample INF_R_359_r3 as a TOP25 hit are listed in **Table S3.** For each model it is noted, whether the drug was within the TOP25 hits (yes) or not (no). For the INF_R_359_r3-derived models, 21 drugs overlapped in all three models.

**Table S3. TOP25 Drug Hits for INF_R_359_r3**

| Drug name | TOP25 FTC | TOP25 LTC | TOP25 mPDX-C |
|---|---|---|---|
| AMG-232 | yes | yes | yes |
| navitoclax | yes | yes | yes |
| vinorelbine | yes | yes | yes |
| bortezomib | yes | yes | yes |
| topotecan | yes | yes | yes |
| idasanutlin | yes | yes | yes |
| talazoparib | yes | yes | yes |
| selinexor | yes | yes | yes |
| mitoxantrone | yes | yes | yes |
| etoposide | yes | yes | yes |
| A-1155463 | yes | yes | yes |
| paclitaxel | yes | yes | yes |
| daunorubicin | yes | yes | yes |
| doxorubicin | yes | yes | yes |
| staurosporine_drug [1] | yes | yes | yes |
| A-1210477 | yes | yes | yes |
| gemcitabine | yes | yes | yes |
| vincristine | yes | yes | yes |
| cytarabine | yes | yes | yes |
| ceritinib | yes | yes | yes |
| panobinostat | yes | yes | yes |
| dactinomycin | yes | yes | no |
| irinotecan | yes | yes | no |
| A-1331852 | no | yes | yes |
| cisplatin | yes | no | yes |
| ponatinib | yes | no | yes |
| volasertib | no | yes | no |
| dasatinib | no | no | yes |

[1] assay control

The comparison of the DNA methylation profile and copy number variations of the neuroblastoma tumor sample INF_R_359_r3 is depicted in **Figure S1.**

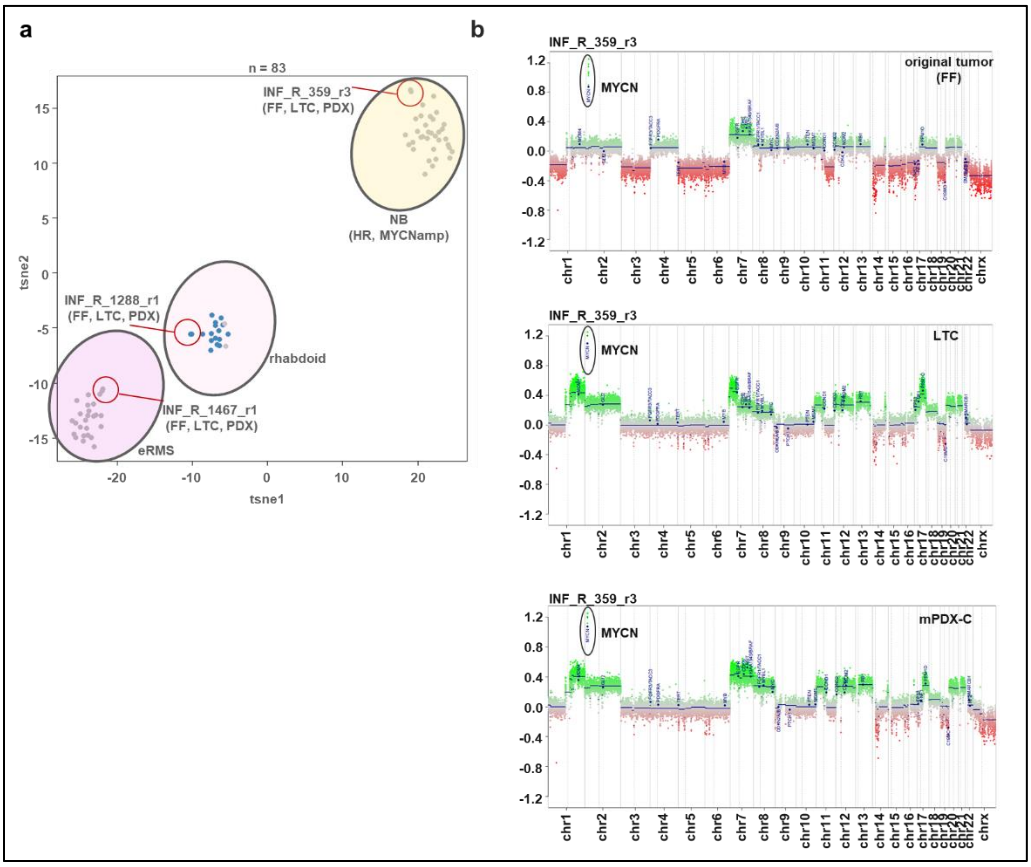


**Figure S1: Comparison of the original tumor with matched culture models. (a)** t-SNE analysis of DNA methylation profiles for comparison of the original tumors and their tumor-derived culture models LTC and mPDX-C with already existing well-characterized reference tumors (rhabdoid tumors; eRMS tumors and high-risk MYCNamp neuroblastomas). **(b)** Copy-number profiles of the original neuroblastoma tumor INF_R_359_r3, and its LTC and mPDX-C models reveal similar genome-wide methylation patterns and recurrent *MYCN* amplification, characteristic for high-risk neuroblastomas. FF: fresh frozen material of the original tumor; LTC: long-term culture; mPDX-C: mouse-PDX-derived culture; NB: neuroblastoma; HR: high-risk.

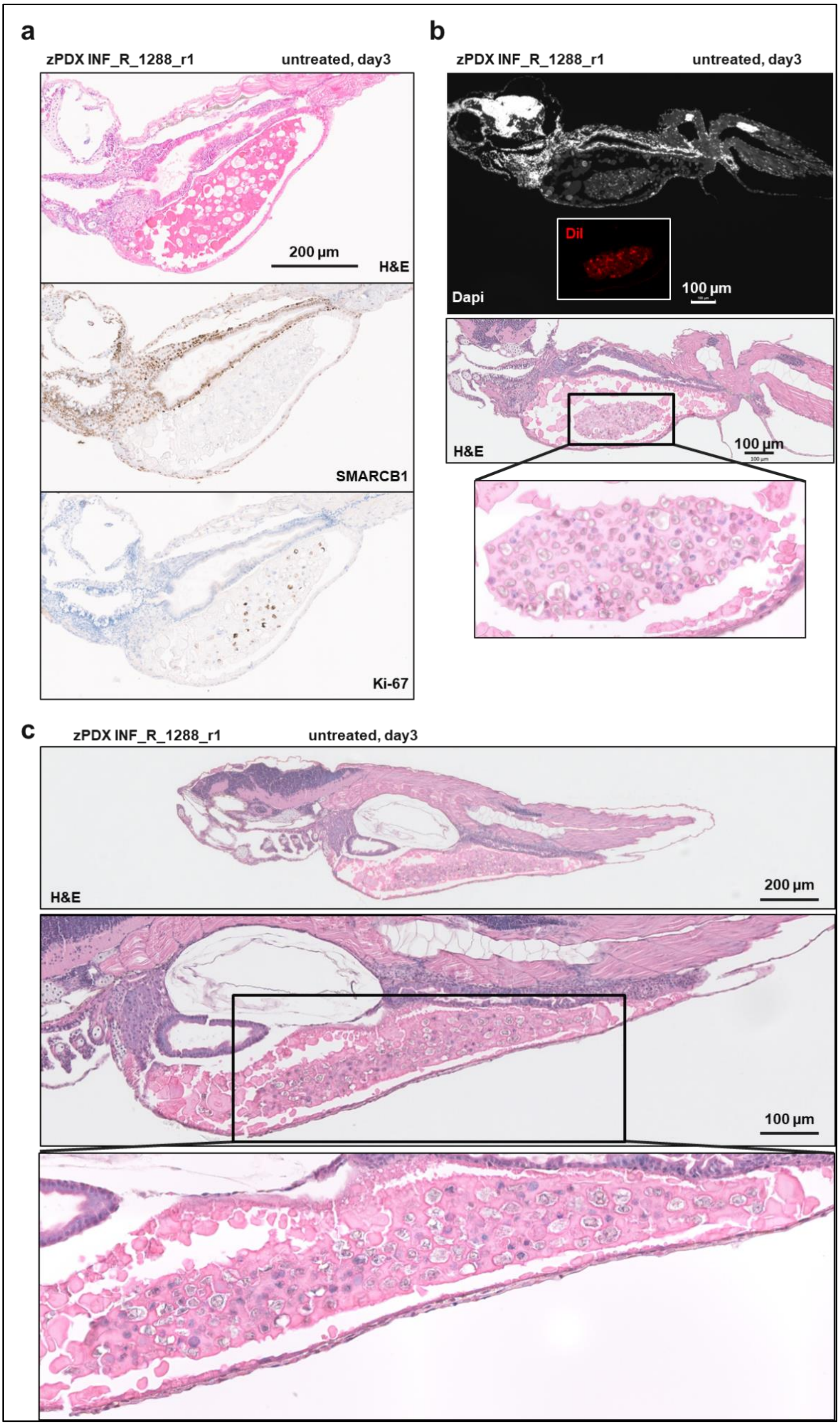


**Figure S2: Representative images of immunohistochemistry (IHC) staining**. **(a)** Zebrafish early larvae engrafted with INF_R_1288_r1 cells (3 days post implantation) were stained with H&E, with $\alpha$-SMARCB1, confirming SMARCB1 deletion in engrafted human tumor cells, and with Ki-67 for the detection of mitotic cells. Scale bar = 200 µm. **(b)** Zebrafish early larvae engrafted with INF_R_1288_r1 cells (3 days post implantation) were stained with DAPI and H&E. Scale bar: 100 µm. **(c)** H&E staining of

zebrafish early larvae engrafted with INF_R_1288_r1 cells (3 days post implantation). Scale bar upper panel: 200 µm, middle panel: 100µm.

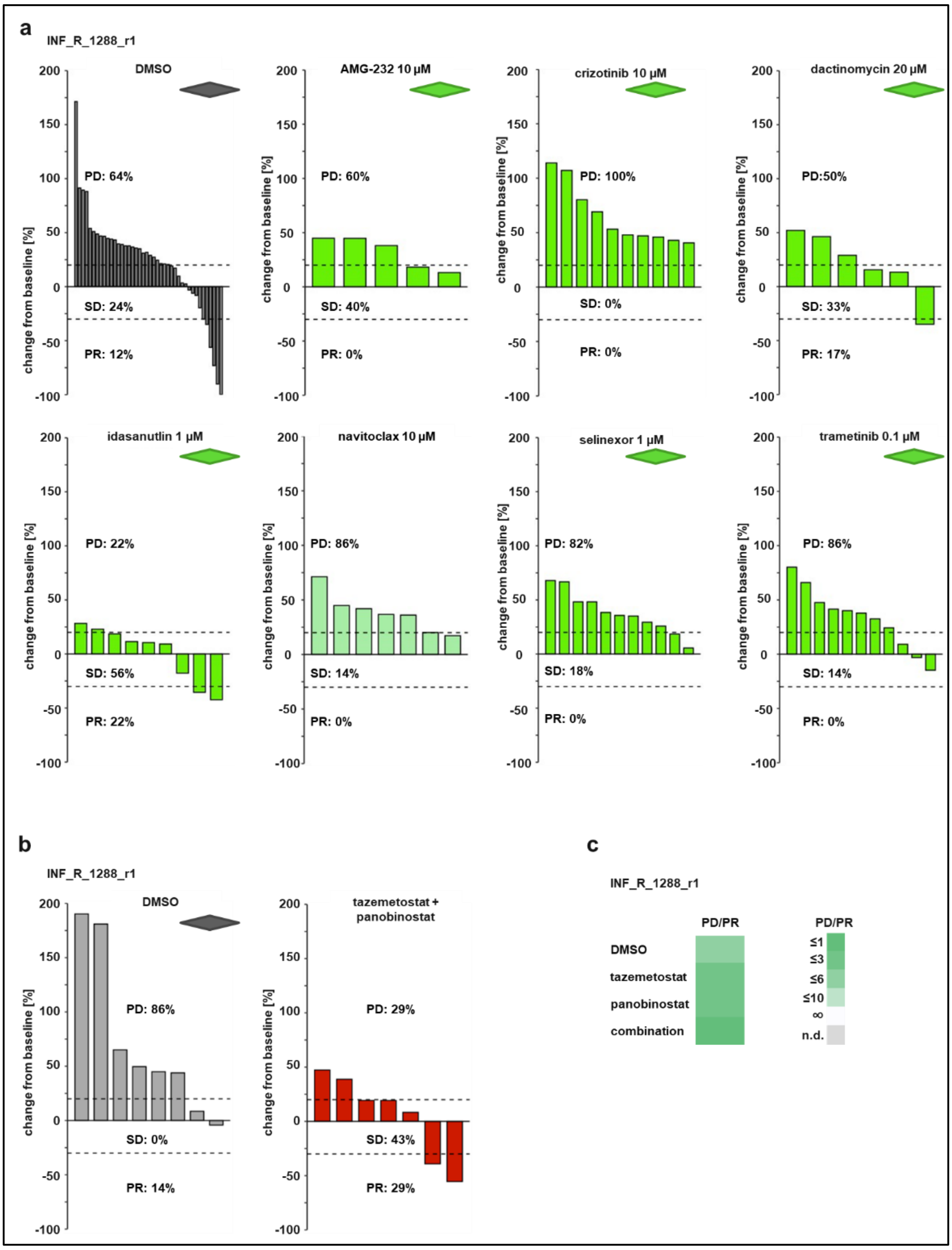


**Figure S3: Waterfall plots demonstrating change in tumor volume for the INF_R_1288_r1 rhabdoid tumor zPDX model. (a—b)** Depicted is the change in tumor volume [%] for each individual zebrafish early larvae engrafted with tumor cells, from baseline (day 1 = start of the treatment) to day 3 after tumor implantation. Numbers indicate the percentage of early larvae with progressive disease (PD), stable disease (SD) and partial response (PR) in each treatment group on day 3. **(c)** Heatmap reflecting the ratio of PD to PR (green shading). PD: progressive disease, tumor volume must have increased at least 20%; PR: partial response, tumor volume must have decreased by more than 30%. n.d.: not detected; ∞: the percentage of PR was 0%.

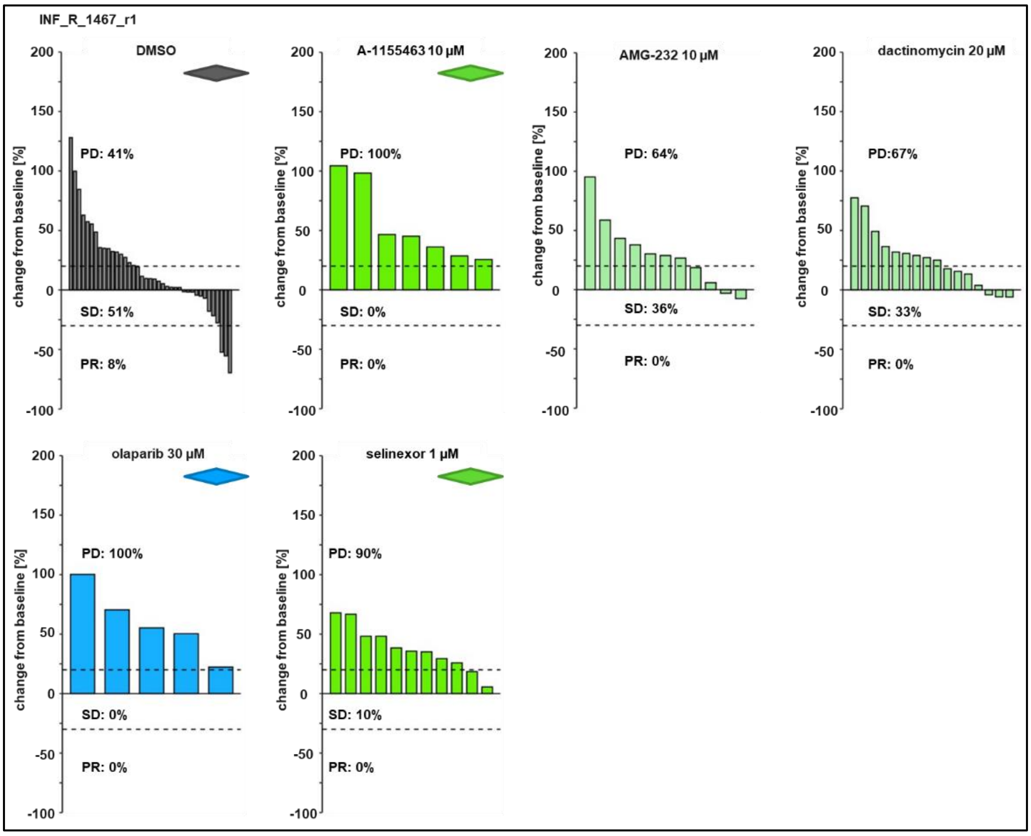


**Figure S4: Waterfall plots demonstrating change in tumor volume for the INF_R_1467_r1 eRMS zPDX model.** Depicted is the change in tumor volume [%] for each individual zebrafish early larvae engrafted with tumor cells, from baseline (day 1 = start of the treatment) to day 3 after tumor implantation. Numbers indicate the percentage of early larvae with progressive disease (PD), stable disease (SD) and partial response (PR) in each treatment group on day 3.

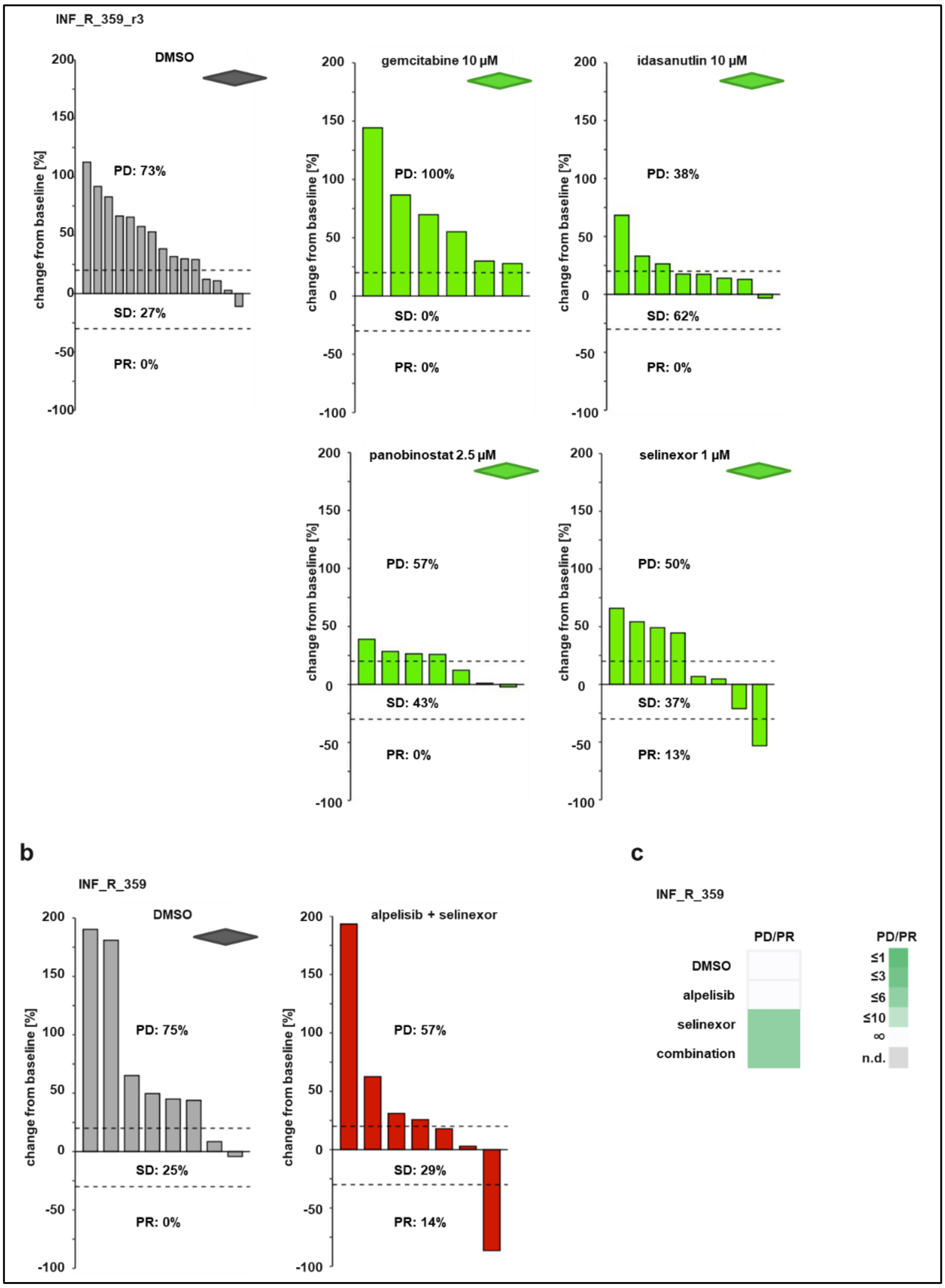


**Figure S5: Waterfall plots demonstrating change in tumor volume for the INF_R_359_r3 neuroblastoma zPDX model. a-b)** Depicted is the change in tumor volume [%] for each individual zebrafish early larvae engrafted with tumor cells, from baseline (day 1 = start of the treatment) to day 3 after tumor implantation. Numbers indicate the percentage of early larvae with progressive disease (PD), stable disease (SD) and partial response (PR) in each treatment group on day 3. **(c)** Heatmap reflecting the ratio of PD to PR (green shading). PD: progressive disease, tumor volume must have increased at least 20%; PR: partial response, tumor volume must have decreased by more than 30%. n.d.: not detected; ∞: the percentage of PR was 0%.
